# Longitudinal amyloid and tau accumulation in autosomal dominant Alzheimer’s disease: findings from the Colombia-Boston (COLBOS) biomarker study

**DOI:** 10.1186/s13195-020-00765-5

**Published:** 2021-01-15

**Authors:** Justin S. Sanchez, Bernard J. Hanseeuw, Francisco Lopera, Reisa A. Sperling, Ana Baena, Yamile Bocanegra, David Aguillon, Edmarie Guzmán-Vélez, Enmanuelle Pardilla-Delgado, Liliana Ramirez-Gomez, Clara Vila-Castelar, Jairo E. Martinez, Joshua T. Fox-Fuller, Claudia Ramos, Martin Ochoa-Escudero, Sergio Alvarez, Heidi I. L. Jacobs, Aaron P. Schultz, Jennifer R. Gatchel, J. Alex Becker, Samantha R. Katz, Danielle V. Mayblyum, Julie C. Price, Eric M. Reiman, Keith A. Johnson, Yakeel T. Quiroz

**Affiliations:** 1 Massachusetts General Hoospital, Harvard Medical School, Boston, MA USA; 2 Grupo de Neurociencias de Antioquia, Universidad de Antioquia, Medellin, Colombia; 3 Brigham and Women’s Hoospital, Harvard Medical School, Boston, MA USA; 4 Boston University, Boston, MA USA; 5 Hospital Pablo Tobón Uribe, Medellín, Colombia; 6 Alzheimer Center Limburg, School for Mental Health and Neuroscience, Maastricht University, Maastricht, Netherlands; 7 Banner Alzheimer’s Institute, Phoenix, AZ USA

**Keywords:** Alzheimer’s, Tau, Amyloid, Imaging, Longitudinal, Autosomal-Dominant

## Abstract

**Background:**

Neuroimaging studies of autosomal dominant Alzheimer’s disease (ADAD) enable characterization of the trajectories of cerebral amyloid-β (Aβ) and tau accumulation in the decades prior to clinical symptom onset. Longitudinal rates of regional tau accumulation measured with positron emission tomography (PET) and their relationship with other biomarker and cognitive changes remain to be fully characterized in ADAD.

**Methods:**

Fourteen ADAD mutation carriers (*Presenilin-1* E280A) and 15 age-matched non-carriers from the Colombian kindred underwent 2–3 sessions of Aβ (11C-Pittsburgh compound B) and tau (18F-flortaucipir) PET, structural magnetic resonance imaging, and neuropsychological evaluation over a 2–4-year follow-up period. Annualized rates of change for imaging and cognitive variables were compared between carriers and non-carriers, and relationships among baseline measurements and rates of change were assessed within carriers.

**Results:**

Longitudinal measurements were consistent with a sequence of ADAD-related changes beginning with Aβ accumulation (16 years prior to expected symptom onset, EYO), followed by entorhinal cortex (EC) tau (9 EYO), neocortical tau (6 EYO), hippocampal atrophy (6 EYO), and cognitive decline (4 EYO). Rates of tau accumulation among carriers were most rapid in parietal neocortex (~ 9%/year). EC tau PET signal at baseline was a significant predictor of subsequent neocortical tau accumulation and cognitive decline within carriers.

**Conclusions:**

Our results are consistent with the sequence of biological changes in ADAD implied by cross-sectional studies and highlight the importance of EC tau as an early biomarker and a potential link between Aβ burden and neocortical tau accumulation in ADAD.

**Supplementary Information:**

The online version contains supplementary material available at 10.1186/s13195-020-00765-5.

## Background

Alzheimer’s disease (AD) is characterized by the presence of amyloid-beta (Aβ) and tau pathologies, which are thought to accumulate for many years during preclinical stages and lead to the neurodegeneration and cognitive decline observed at the clinical phase [1–3]. Mutations of the Presenilin -1 (*PSEN1*; OMIM 104311) gene predispose individuals to develop autosomal dominant Alzheimer disease (ADAD) in early adulthood [4], showing pathologic and neurodegenerative changes similar to those seen in late onset or sporadic AD [5–7]. Studies of extended families with ADAD, including the Colombian kindred with approximately 6000 living members and an estimated 1200 *PSEN1* E280A (Glu280Ala) mutation carriers, have enabled characterization of the trajectory of AD-related biological and behavioral changes in the decades prior to clinical symptom onset [8–10]. Ongoing studies of these families continue to inform natural history studies and prevention therapeutic trials for AD [11].

Positron emission tomography (PET) has enabled the in vivo characterization and serial tracking of Aβ and tau accumulation [12–16]. Findings from tau PET studies of sporadic AD have been consistent with the spatiotemporal progression of tauopathy implied by postmortem studies [17]; namely, that cortical tau accumulation begins focally in medial temporal lobe (MTL) allocortex and later spreads to temporal and extratemporal neocortex (also called “isocortex”) in association with Aβ [12, 13]. Whether tau accumulation follows a similar spatiotemporal pattern in ADAD remains unresolved [18, 19]. We previously reported that abnormally elevated MTL (entorhinal cortex, EC) tau PET was evident in *PSEN1* E280A carriers relative to non-carriers from the Colombian kindred as early as 6 years prior to expected onset of mild cognitive impairment (MCI), and the overall spatial pattern of tau PET in ADAD was similar to that seen in sporadic AD [18]. Another ADAD study found that tau PET was elevated only among impaired individuals, and suggested that MTL tauopathy including EC may be less involved in ADAD compared to sporadic AD [19].

Longitudinal PET studies have demonstrated pathological changes in vulnerable sporadic AD populations [20–24], but to our knowledge, longitudinal measures of both tau and Aβ PET in conjunction with measures of brain structure and cognitive function have not yet been reported in ADAD. We tracked changes over a 2–4-year period in PET measures of tau and Aβ, magnetic resonance imaging (MRI) measures of brain structure, and cognitive assessments in young *PSEN1* E280A mutation carriers and age-matched non-carriers from the Colombian ADAD kindred. We evaluated differences in biomarker change rates between carriers and non-carriers, as well as associations between age, rates of Aβ and tau accumulation, neurodegeneration, and cognitive decline. We hypothesized that these longitudinal measures would reveal a sequence of changes in ADAD beginning with Aβ accumulation, followed by EC tau, neocortical tau, neurodegeneration, and cognitive decline.

## Methods

### Participants and procedures

Eleven cognitively unimpaired *PSEN1* mutation carriers, three cognitively impaired mutation carriers, and fifteen age-matched non-carriers from the Massachusetts General Hospital (MGH) COLBOS (Colombia-Boston) longitudinal biomarkers study participated in this study (Table 1). Participants were recruited from the Alzheimer Prevention Initiative (API) registry. Exclusion criteria included a diagnosis of dementia, a significant medical, psychiatric, or neurological disorder, or a history of stroke, seizures, substance abuse, or other disorders affecting motor, visuospatial, or cognitive abilities. Cognitively unimpaired individuals had to demonstrate no cognitive impairment on the Consortium to Establish a Registry for Alzheimer’s Disease neuropsychological battery (CERAD) word list recall and visuospatial memory tests, a Mini-Mental State Examination (MMSE) score of 26 or greater, a clinical diagnostic rating scale (CDR) score of 0, and a Functional Assessment Staging Test (FAST) score of 2 or less, to be included in this study. Individuals with mild cognitive impairment (MCI) were diagnosed based on Petersen et al.’s (2014) criteria [25]. which includes subjective cognitive concerns, mild impairment in memory tests, intact activities of daily living, and FAST score of 3. The expected years to onset (EYO) were calculated by subtracting carriers’ ages from 44, the median age of clinical onset in *PSEN1* E280A carriers (95% CI = 43–45) [26].

**Table 1:** Sample characteristics

	Non-carriers	Unimpaired Carriers	Impaired carriers	Full sample
**N**	15	11	3	29
**Imaging sessions** Baseline | 18-24mo. | 42-48mo.	15 | 13 | 4	11 | 11 | 5	3 | 2 | 1	29 | 26 | 10
**Cognitive testing sessions** Baseline | 18-24mo. | 42-48mo.	15 | 15 | 4	11 | 10 | 5	3 | 3 | 1	29 | 28 | 10
**Total follow-up time (Y)**	2.33 ± 0.97 [1.61-4.33]	2.82 ± 1.09 [1.85-4.33]	2.40 ± 1.05 [1.61-3.59]	2.52 ± 1.01 [1.61-4.33]
**Baseline age (Y)**	39.2 ± 5.9 [28.5-50.2]	37.3 ± 5.0 [28.5-43.5]	44.5 ± 1.9 [42.5-46.2]	39.0 ± 5.6 [28.5-50.2]
**Females, N (%)**	10 (66.7)	7 (63.6)	3 (100.0)	20 (69.0)
**Education (Y)**	9.3 ± 4.4 [2-16]	9.5 ± 4.8 [2-17]	13.3 ± 2.5 [11-16]	9.8 ± 4.5 [2-17]
**Baseline MMSE**	29.3 ± 0.7 [28-30]	28.0 ± 1.2 [26-30]	24.3 ± 5.5 [18-28]	28.3 ± 2.3 [18-30]

Continuous variables are given as mean ± standard deviation [range]. *Y* years, *MMSE* Mini Mental State Examination score

All participants traveled from Colombia to Boston every 18 to 24 months to undergo MRI and amyloid and tau PET imaging at MGH. Neuropsychological (NP) evaluations were conducted in Spanish at the University of Antioquia within 6 months of imaging. Table 1 shows demographic and study timeline information for all participants. A total of 26 participants had PET, MRI, and NP sessions at baseline and 18–24-month follow-up, and 10 of these had an additional follow-up time point at 42–48 months. Three participants had baseline imaging and NP but only completed NP at 18–24 months (due to inability to travel to Boston for follow-up imaging); these were included in analyses of baseline imaging vs. cognitive change.

All participants provided informed written consent prior to enrollment and were studied under guidelines approved by local institutional review boards of the University of Antioquia in Colombia and the Massachusetts General Hospital in Boston. Investigators and participants were blinded to the participants’ *PSEN1* E280A carrier status.

### MRI

Structural T1-weighted data were acquired using a Siemens 3 Tesla Tim Trio (Siemens, Erlangen, Germany; repetition time = 2300 ms, echo time = 2.95 ms, flip angle = 9°, and a voxel size = 1.05 × 1.05 × 1.2 mm. Images were processed with FreeSurfer (FS) v6.0 (http://surfer.nmr.mgh.harvard.edu) to identify white and pial surfaces, standard regions-of-interest (ROI) from the Desikan atlas for PET sampling, and hippocampal volumes (HV) [27]. FS outputs were quality checked and manually edited where necessary to ensure accurate segmentation and surface identification. HV measures derived from FS were adjusted for intracranial volume (ICV) by regressing out the contribution of ICV on HV, using previously published parameters [28].

### PET

18F-flortaucipir (FTP) and 11C-Pittsburgh compound B (PiB) were prepared and acquired according to previously published protocols [12]. All PET data were acquired on a Siemens ECAT HR+ (3D mode; 63 image planes; 15.2 cm axial field of view; 5.6 mm transaxial resolution; and 2.4 mm slice interval). PiB data were acquired using a 60-min dynamic protocol and analyzed by the Logan reference method with distribution volume ratio (DVR) as outcome. FTP data were acquired from 80 to 100 min post-injection in 4 × 5-min frames with the standardized uptake value tissue ratio (SUVR) as outcome. Cerebellar gray matter was used as reference for PiB and FTP. Partial volume correction (PVC) was applied to the PET frame data using geometric transfer matrix (GTM) method for PiB and an extended Muller–Gartner method (implemented in FS) for FTP [29], with estimated 6 mm full-width at half-maximum (FWHM). We also assessed PET data without PVC for comparison.

PET images were affine co-registered to each subject’s contemporaneous T1 images (SPM8) and all PET data sets were sampled using FS-derived ROIs. Aβ burden was represented using PiB DVR in a large neocortical target region that included frontal, lateral temporal and parietal, and retrosplenial cortices (FS-defined FLR region) [12]. These neocortical PiB values were standardized to an approximate Centiloid (CL) scale [30] using a previously published conversion in this pipeline [15] to facilitate comparison with other studies. A previously published PiB PVC FLR DVR threshold of 1.32 (~ 19 CL) was used to indicate elevated Aβ burden [15]. For comparison with other studies, rates of PiB change were characterized in all ROIs from the Desikan atlas as well as striatum (volume-weighted average of bilateral caudate and putamen), which may represent an earlier stage of Aβ accumulation in ADAD [31, 32].

Based on previous neuropathology [17] and PET data [12, 13, 23], FTP uptake was assessed primarily in three ROIs from the Desikan atlas: entorhinal (EC), inferior temporal (IT), and precuneus (PC) cortices, representing medial temporal lobe (MTL) allocortex, temporal neocortex, and extra-temporal neocortex, respectively. These three ROIs were used for statistical analyses, described below. SUVR thresholds for elevated tau in each ROI were defined as two standard deviations above the mean in non-carriers (EC: 1.26, IT: 1.42, PC: 1.30). For comparison with other studies, we also reported rates of FTP change for all Desikan atlas ROIs as well as rhinal cortex (RC), a PET-compatible measure of the trans-entorhinal region [15]; a composite ROI (unweighted SUVR average) consisting of bilateral entorhinal, amygdala, lateral occipital, and inferior temporal (EAOT), which has been shown to effectively discriminate subjects with low- and high-FTP uptake [33]; and a temporal lobe composite ROI (volume-weighted average of entorhinal, amygdala, parahippocampal, fusiform, inferior and middle temporal) suggested by another longitudinal tau PET study [20].

**Fig. 1:**
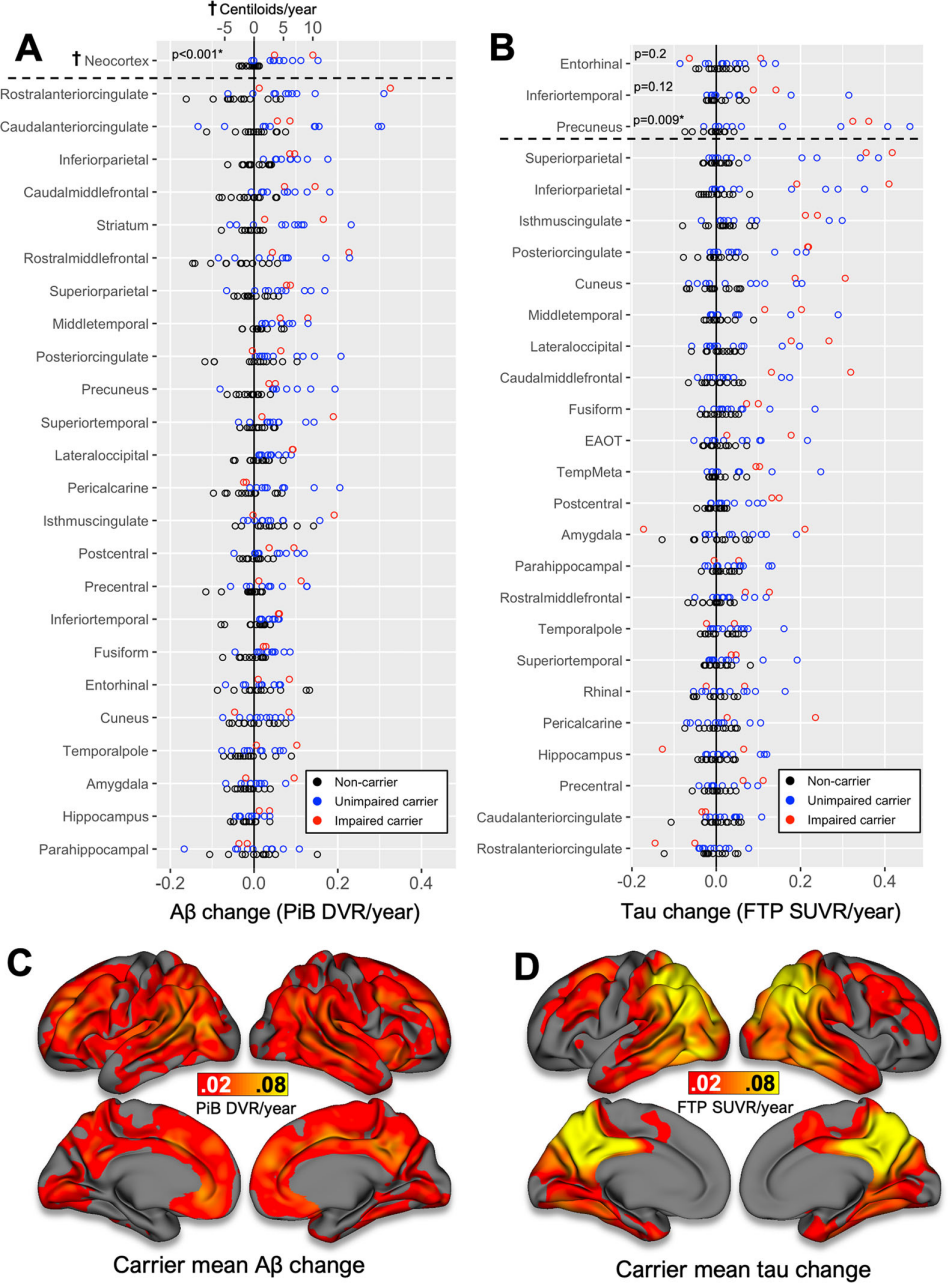
*PSEN1* E280A mutation carriers show greater rates of tau and Aβ increase compared to non-carriers. *Upper*, dot plots of Aβ (**a**, left) and tau (**b**, right) PET change rates, expressed as annualized change in PiB DVR and FTP SUVR respectively, in all regions of interest (ROIs). Dots are color-encoded by carrier and cognitive status according to inset legend (lower right). Primary ROIs for each modality are shown above horizontal dashed line; other ROIs below the dashed line are ordered from top to bottom by highest to lowest mean change rate within all carriers. Neocortical Aβ change rates were normalized to the Centiloid scale, shown at top (**†**); note that the Centiloid scale does not apply to any other ROI. Adjusted *p* values for group difference (Mann-Whitney, Bonferroni-Holms correction) between all carriers and non-carriers are given for primary ROIs (*p < 0.05 after multiple comparisons correction); other regions are shown for comparison with other studies but were not included in statistical analyses. EAOT = aggregate (unweighted average) of bilateral entorhinal, amygdala, occipital, and inferior temporal ROIs (Mishra et al. [33]); TempMeta = aggregate of bilateral entorhinal, amygdala, parahippocampal, fusiform, inferior and middle temporal (Jack et al. [20]). *Lower*, Surface visualization of mean rates of Aβ (**c**, left) and tau (**d**, right) change within all carriers, expressed as annualized change in PiB DVR and FTP SUVR, respectively, according to the color bar

For visualization purposes, FTP SUVR and PiB DVR images were normalized to standard (MNI) space and projected onto the fsaverage surface using FS methods (sampled at the midpoint of gray matter, surface-smoothed 8 mm). Mean rates of PiB and FTP change within carriers were computed vertex-wise and visualized on the cortical surface (Fig. 1c, d). Mean images of FTP SUVR and PiB DVR for each group at baseline and 2-year follow-up are shown in Supplementary Figure 1.

### Neuropsychological evaluations

Participants underwent a comprehensive NP battery at each time point, including the Spanish-language version of the Consortium to Establish a Registry for Alzheimer’s Disease (CERAD) battery, which has been validated for the assessment of memory, language, and praxis in the Colombian kindred [34], and all the measures comprising the Preclinical Alzheimer’s Cognitive Composite (PACC) [35], which was designed to assess performance across episodic memory, executive function, and orientation. Specifically, PACC scores were computed using the following measures: Free and Cued Selective Reminding Test (FCSRT) Total Recall score (range 0–48) [36]; Logical Memory Delayed Recall score (0–25) [37], Digit-Symbol Substitution Test score (0–93) [37], and Mini-Mental State Examination (MMSE) total score (0–30) [38]. Raw scores were *z*-transformed using the non-carriers’ baseline mean and standard deviation and averaged to produce PACC *z*-scores, which were used in subsequent statistical analyses. Our primary NP outcome measures were PACC and CERAD word list learning (WLL) scores (0–10), as CERAD WLL has been previously reported to be an early indicator of cognitive decline in this kindred [34].

### Statistics

Annualized change rates were calculated for all imaging and NP variables by extracting slopes from linear regression models of each variable as a function of time from baseline (in years) within each participant. These change rates were used in subsequent statistical analyses, described below. To ensure that biomarker change rates were not systematically biased by the use of individual regression models, we also confirmed our main findings using slopes derived from linear mixed-effects models (Supplementary Figure 2). For the purposes of interpretability and comparison with other studies, we also reported rates of change as annualized percentage change, defined as the annualized change rate (as described above) divided by the baseline value and multiplied by 100.

Group differences between carriers and non-carriers were assessed with Mann-Whitney *U* tests, and bivariate relationships among baseline and change measures using Spearman correlations. Regional differences in rates of tau accumulation (between EC, IT, and PC) among carriers were evaluated using paired Wilcoxon tests. We used a Bonferroni-Holm correction for multiple comparisons in analyses that included tau variables to mitigate the possibility of false positives from testing multiple tau ROIs. Given previous reports showing a sigmoidal trajectory of Aβ accumulation [39], we tested for a quadratic relationship between baseline Aβ and Aβ change rates in the full sample by assessing model fits of baseline versus change with and without a quadratic term. For comparison, we also assessed the relationship between baseline tau and tau change rates in the same way. We report the *r*^2^ estimates with standard error (SE) and *p* values for these models. All statistical analyses, including group comparisons, Spearman correlations, and regressions, were performed in R (version 3.4.1).

## Results

### *PSEN1* mutation carriers show faster Aβ accumulation rates compared to non-carriers

**Fig. 2:**
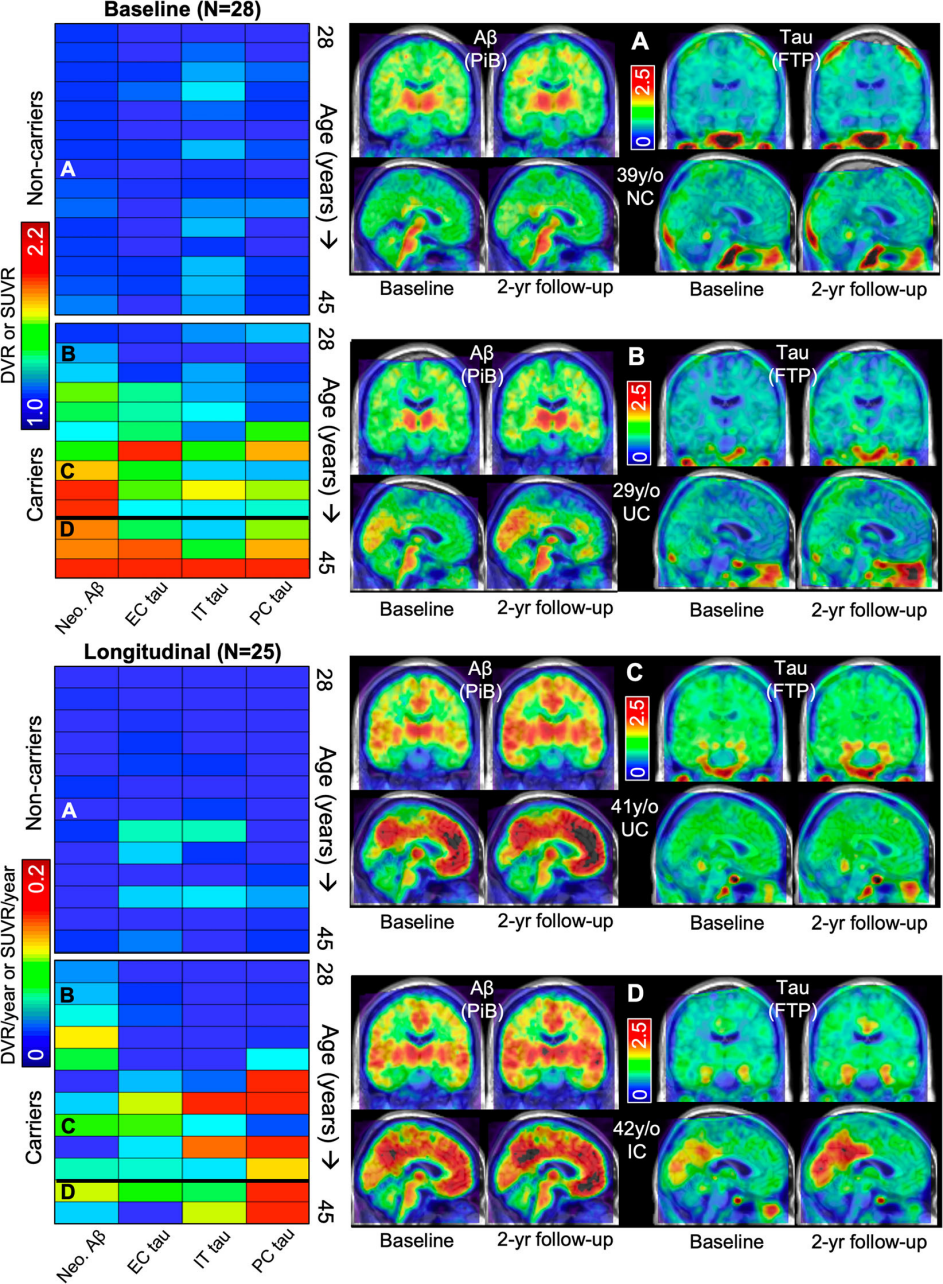
Longitudinal Aβ and tau PET images in *PSEN1* E280A carriers. *Top left*: Matrix showing baseline Aβ (PiB DVR) and tau (FTP SUVR) PET measures (color bar) for all subjects: Each column is a PET variable, and each row is a subject, separated by *PSEN1* E280A carrier status and ordered by age increasing from top to bottom (right labels; expected age at symptom onset in carriers = 44 years, 95% CI [43–45]). *Bottom left*: Matrix showing annualized Aβ and tau PET change rates (color bar) for all subjects, arranged as above. Horizontal thick black line separates cognitively unimpaired carriers (UC, above line) from cognitively impaired carriers (IC, below line). We assessed Aβ burden in a large neocortical aggregate (Neo.) and tau burden in three primary ROIs: entorhinal cortex (EC), inferior temporal gyrus (IT), and precuneus (PC). Four exemplary cases are labeled (A–D), with corresponding baseline and 2-year follow-up PET slice data shown at right. A–D: Aβ (PiB DVR, left) and tau (FTP SUVR. right) PET images at baseline and 2-year follow-up in coronal (upper) and sagittal (lower) slices for four exemplary cases, labeled in color matrices at left. Age and carrier status are given for each participant (y/o, years old; NC, non-carrier; UC, unimpaired carrier; IC, impaired carrier)

Compared with age-matched non-carriers, *PSEN1* E280A mutation carriers showed significantly faster rates of Aβ accumulation (Mann-Whitney *p* < 0.001 for neocortex, Figs. 1 and 2). Rates of Aβ change were similar for unimpaired and impaired carriers (Fig. 1a), averaging (mean ± SD) 0.07 ± 0.05 DVR/year in neocortex (4.0 ± 2.7%/year; 4.8 ± 3.5 CL/year). Regionally, the highest rates of Aβ change among carriers were observed in anterior cingulate, lateral parietal, and middle frontal cortices (Fig. 1a, c).

**Fig. 3:**
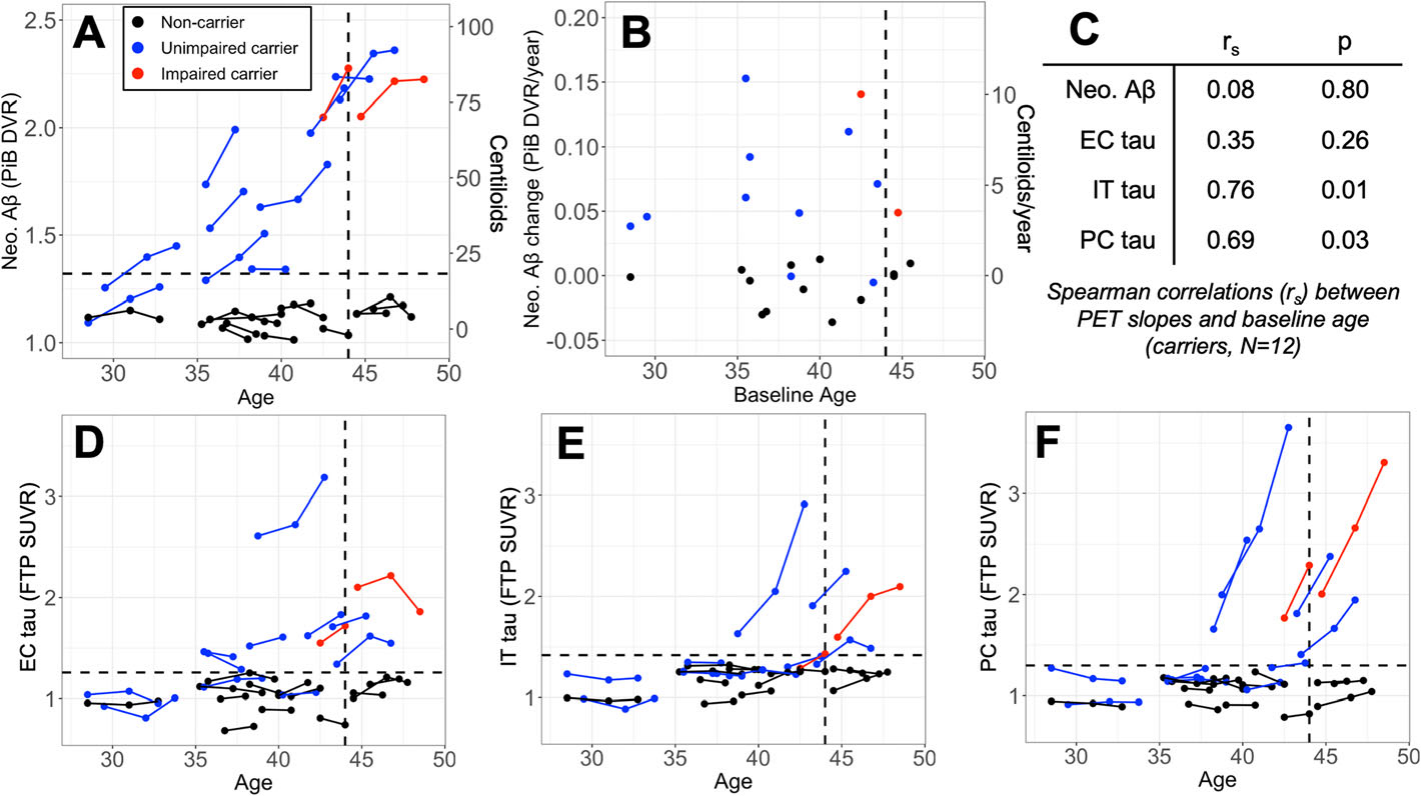
Steady Aβ accumulation precedes rapid neocortical tau increase in *PSEN1* E280a mutation carriers. Spaghetti plots show Aβ (*top*, **a**) and tau (*bottom*, **d–f**) PET levels in ROIs vs. age at baseline and 2–4-year follow-up; scatter plot (**b**) shows rates of Aβ accumulation vs. baseline age. Aβ PET (PiB DVR) was assessed in a neocortical aggregate (Neo., **a–b**); Neo. Aβ levels were normalized to an approximate Centiloid (CL) scale, shown in A-B at right; horizontal dashed line in **a** indicates previously-published high-PiB threshold DVR = 1.32 (15). Tau PET (FTP SUVR) was assessed in entorhinal (EC, **d**), inferior temporal (IT, **e**), and precuneus (PC, **f**) cortices. Vertical dashed line indicates expected age of cognitive symptom onset (44 years); horizontal dashed lines in (**d–f**) indicate two standard deviations above the mean FTP SUVR in non-carriers (EC: 1.26, IT: 1.42, PC: 1.30). Dots and lines are colored by subject group according to inset legend (top, center). **c** Gives the Spearman correlations between age and annualized change rates (i.e., slopes) in each PET variable (rows) within carriers (*N* = 12) with *p* values after adjustment for multiple comparisons

While age and Aβ burden were highly correlated cross-sectionally (*r*_*s*_ = 0.93, *p* < 0.001), longitudinal rates of Aβ accumulation were not associated with baseline age among carriers (Fig. 3b, c, *p* = 0.8). One of the youngest carriers in our sample (15–20 EYO) showed baseline Aβ burden comparable to non-carriers (1.09 neocortical DVR, ~ 2 CL), but accumulated neocortical Aβ over 4 years at a faster rate compared to non-carriers (0.04 DVR/year ~ 2.7 CL/year; 3.5%/year), consistent with previous work suggesting that neocortical Aβ increases beginning more than a decade prior to symptom onset in carriers. Supplementary analyses of striatum and precuneus Aβ indicated that these regional measures may be elevated at younger age compared to the global measure (Supplementary Figure 3).

**Fig. 4:**
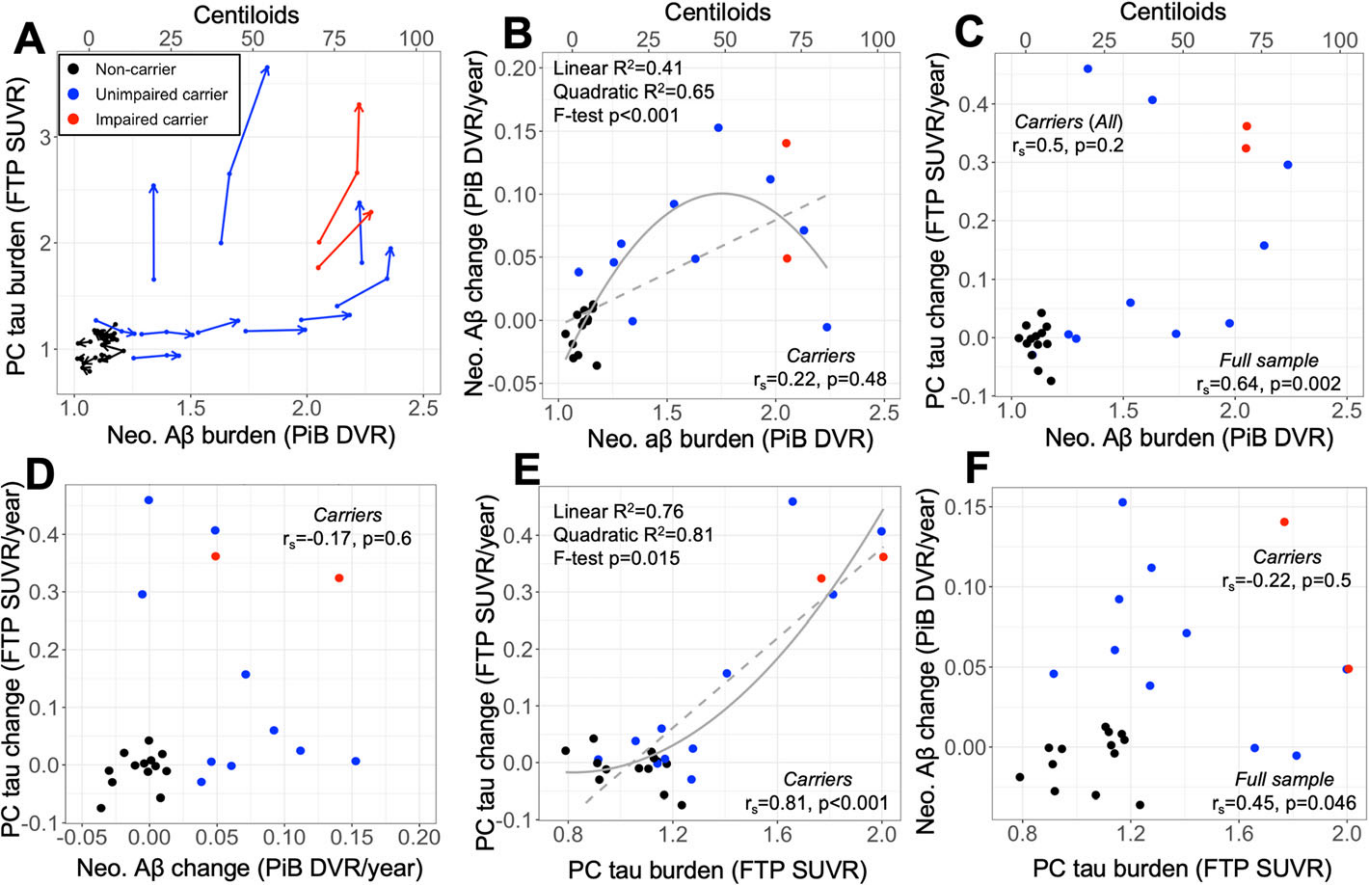
Contemporaneous rates of Aβ and tau accumulation are not correlated among *PSEN1* E280a mutation carriers. **a** Longitudinal trajectories of tau and Aβ burden, expressed as precuneus (PC) Flortaucipir (FTP) standardized uptake value ratio (SUVR) and neocortical (Neo.) Pittsburgh Compound B (PiB) distribution volume ratio (DVR), respectively. Lines and dots are colored by subject group according to inset legend, and arrowhead indicates most recent follow-up time point. **b** Relationship between baseline Aβ and Aβ change rate, which was quadratic in the full sample (inset text, top left). Linear and quadratic regression fits are shown as dashed and solid gray curves, respectively. **c** Relationship between baseline Aβ and PC tau change rates. **d** Relationship between Aβ and PC tau and change rates, expressed as annualized PiB DVR or FTP SUVR change, respectively. **e** Baseline PC tau vs PC tau change rate. Linear and quadratic regression fits are shown as dashed and solid gray curves, respectively. **f** Baseline PC tau vs Aβ change rate

In the full sample (including carriers and non-carriers), the relationship between baseline Aβ and Aβ change was quadratic (Fig. 4b) with a negative quadratic term (β (SE) = − 0.25 (0.06), *p* < 0.001), consistent with a sigmoidal trajectory of Aβ accumulation.

### Rapid neocortical tau accumulation is observed in carriers near time of symptom onset

Longitudinal rates of regional tau accumulation are shown in Fig. 1b. While we observed considerable variability among carriers in rates of tau accumulation, carriers on average had higher mean rates of tau change in PC (0.16 ± 0.18 SUVR/year; 9.3 ± 9.8%/year) compared to IT (0.06 ± 0.10 SUVR/year; 3.8 ± 6.0%/year, paired Wilcoxon test *p* = 0.03) and EC (0.03 ± 0.07 SUVR/year; 1.8 ± 4.0%/year, *p* = 0.04). Group differences in tau change rates between all carriers and non-carriers were significant for PC (Mann-Whitney *p* = 0.009), but not for IT (*p* = 0.12) or EC (*p* = 0.4). The highest overall mean rates of tau change among all carriers were observed in inferior and superior parietal, followed by PC and isthmus and posterior cingulate cortices (Fig. 1b, d).

Baseline age was associated with greater tau change rates in IT and PC (Fig. 3c). These associations were driven by 4 older carriers in IT and 6 in PC who showed rapid tau increase (i.e., > 0.1 SUVR/year) around the time of symptom onset (39–45 years, Figs. 1 and 3e, f). By contrast, EC tau change rates were less rapid and not associated with baseline age (Fig. 3c, d, *p* = 0.26), and elevated EC tau levels (relative to non-carriers) were observed as early as age 35 (9 EYO), compared to age 38 (6 EYO) in IT and PC (Figs. 2 and 3d).

Baseline tau measures were significantly associated with subsequent tau change rates within neocortical ROIs (IT: *r*_*s*_ = 0.69, *p* = 0.034; PC: *r*_*s*_ = 0.81, *p* = 0.006), but not in EC (*r*_*s*_ = 0.40, *p* = 0.20). In contrast with Aβ, the relationship between baseline tau and tau change rate in PC was significantly quadratic with a positive quadratic term (Fig. 4e; β (SE) = 0.33 (0.12), *p* = 0.015), suggesting that neocortical tau accumulation did not reach a plateau at later stages. Across ROIs, the baseline EC tau level predicted subsequent tau change rates in IT (*r*_*s*_ = 0.80, *p* = 0.018) and PC (*r*_*s*_ = 0.76, *p* = 0.03), whereas baseline IT and PC tau levels did not predict subsequent EC tau change rates (*p* > 0.2), consistent with EC tau accumulation preceding neocortical tau accumulation.

### Aβ rise precedes rapid neocortical tau increase in ADAD

Individual trajectories of Aβ and tau PET within carriers (shown for neocortical Aβ and PC tau in Fig. 4a) were consistent with the accumulation of Aβ leading up to the accumulation of neocortical tau at elevated levels of Aβ. The level of neocortical Aβ at which rapid PC tau increase was observed varied: of six carriers who showed the greatest increases in PC tau, four were older (42–44 years old, 0–2 EYO) and had baseline neocortical PiB DVR > 2.0 (~ 65 CL), while the two with the fastest PC tau accumulation rates (> 0.4 SUVR/year) were younger (both 38 years old = 6 EYO) and had more moderate levels of Aβ (1.63 and 1.34 DVR, ~ 40 and 20 CL, Fig. 4a, c).

Associations between baseline Aβ and subsequent tau accumulation rates within carriers were in the expected direction but not significant after multiple comparisons correction (PC: *r*_*s*_ = 0.50, *p* = 0.20, Fig. 4c; IT: *r*_*s*_ = 0.62, *p* = 0.12; EC: *r*_*s*_ = 0.31, *p* = 0.33). Supplementary analysis of striatum and precuneus PiB revealed that striatum PiB was a stronger predictor of subsequent neocortical tau accumulation compared to either precuneus or global PiB (Supplementary Figure 4). Baseline tau levels were not associated with subsequent Aβ change rates (Fig. 4f; *p* > 0.3 for all tau ROI) within carriers. Tau and Aβ change rates were not correlated among carriers (Fig. 4d; *p* > 0.4 for all tau ROIs), consistent with an asynchronous relationship between Aβ and tau accumulation.

### Hippocampal volume loss occurs in temporal proximity to tau accumulation

**Fig. 5:**
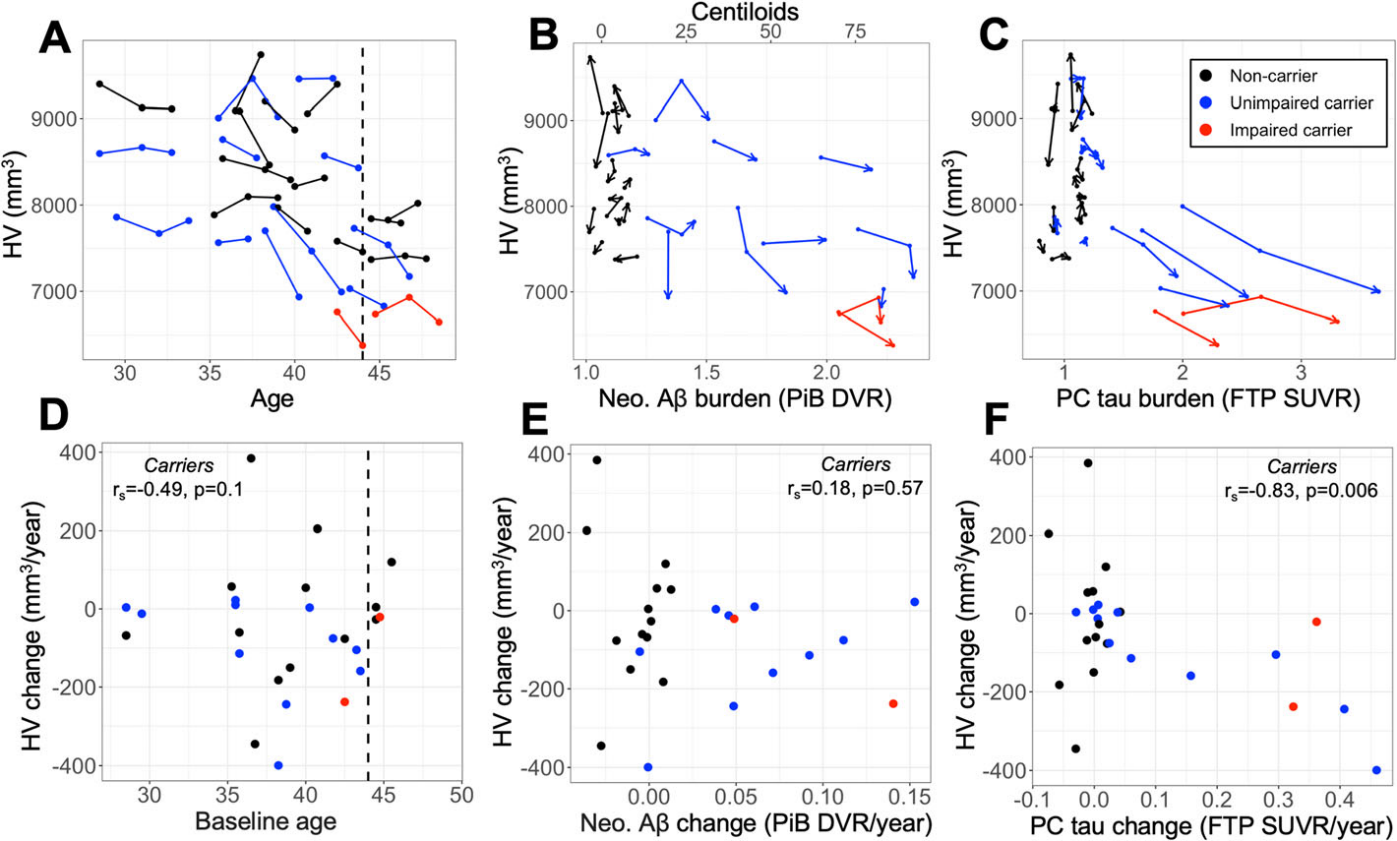
Hippocampal volume loss occurs in temporal proximity to tau accumulation. *Top*, longitudinal trajectories of hippocampal volume (HV, adjusted for intracranial volume) by **a** age, **b** neocortical (Neo.) Aβ burden, and **c** precuneus (PC) tau. Arrowheads in **b**, **c** indicate most recent time point; lines are color-encoded by *PSEN1* E280a carrier and clinical status according inset legend at top-right. *Bottom*, Relationships between annualized change in HV and **d** age, **e** neo. Aβ change rate, and **f** PC tau change rate. Inset text gives Spearman correlation and *p* value within carriers. HV change was marginally associated with and significantly associated with PC tau change rate, but not neo. Aβ change rate, suggesting that loss of HV occurs in temporal proximity to tau accumulation

Relationships between HV, age, and PET measures are shown in Fig. 5. Differences in baseline HV and rates of HV atrophy between carriers and non-carriers were in the expected direction but not significant (HV baseline: mean (SD) = 7900 (850) vs. 8400 (650) mm^3^, *p* = 0.11; HV slope: − 110 (130) vs. − 7 (180) mm^3^/year, *p* = 0.15, Fig. 5d). Within carriers, age was significantly associated with lower baseline HV (*r*_*s*_ = − 0.57, *p* = 0.03) and marginally associated with steeper HV slope (*r*_*s*_ = − 0.49, *p* = 0.1, Fig. 5a). HV slopes were not significantly associated with baseline Aβ (*r*_*s*_ = − 0.29, *p* = 0.35) or baseline tau burden after multiple comparisons correction (PC: *r*_*s*_ = − 0.56, *p* = 0.12; EC baseline: *r*_*s*_ = − 0.46, *p* = 0.26; IT baseline: *r*_*s*_ = − 0.31, *p* = 0.33). Similarly, HV slopes were not associated with Aβ slopes (*r*_*s*_ = 0.18, *p* = 0.57, Fig. 5e) among carriers. By contrast, HV slopes were significantly associated with tau change rates in PC (*r*_*s*_ = − 0.83, *p* = 0.006, Fig. 5f) and marginally associated with EC and IT tau change rates (EC: *r*_*s*_ = − 0.54, *p* = 0.08; IT: *r*_*s*_ = − 0.64, *p* = 0.06) within carriers, suggesting that HV loss occurs closer in time to neocortical tau PET than to Aβ accumulation.

### Compared to longitudinal Aβ accumulation rates, tau accumulation rates are more closely related to cognitive decline

**Fig. 6:**
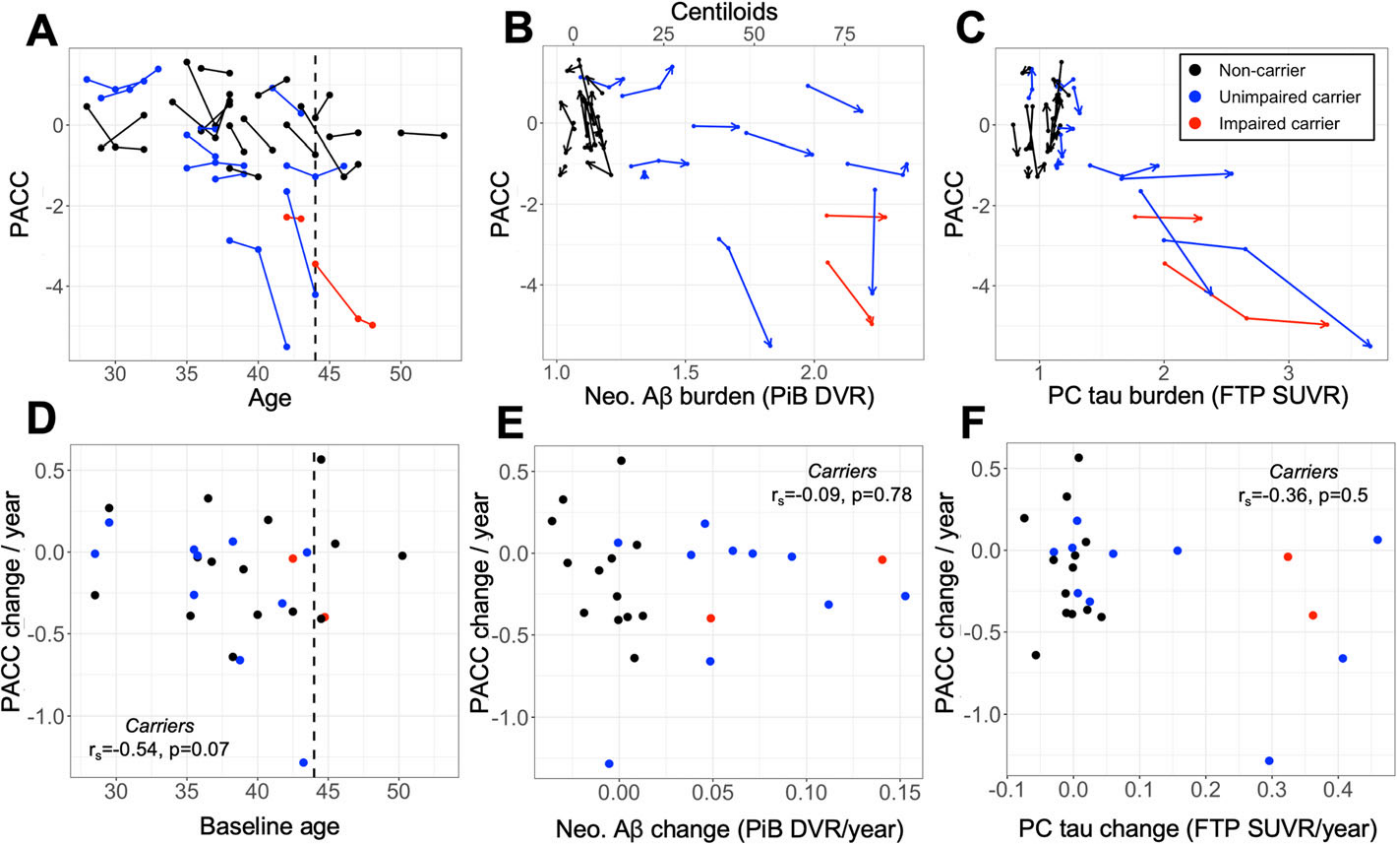
PACC captures cognitive decline in carriers near expected symptom onset. *Top*, longitudinal trajectories of Preclinical Alzheimer’s Cognitive Composite (PACC) score by **a** age, **b** neocortical (Neo.) Aβ burden, and **c** precuneus (PC) tau. Arrowheads in **b**, **c** indicate most recent time point; lines are color-encoded by *PSEN1*-E280a carrier and clinical status according to the inset legend at top-right. *Bottom*, Relationships between annualized change in PACC and **d** age, **e** neo. Aβ change rate, and **f** PC tau change rate. Inset text gives Spearman correlation and *p* value within carriers. PACC change rate showed negative but non-significant associations with age and PC tau change rate, and no association with neo. Aβ change rate

Individual trajectories of cognitive decline as measured by PACC score are shown in Fig. 6a. Rates of PACC decline were marginally associated with greater age within carriers (*r*_*s*_ = − 0.54, *p* = 0.07, Fig. 6d) and the steepest rates of cognitive decline were observed in unimpaired carriers approaching the time of expected symptom onset (baseline 39–43 years, 1–5 EYO). All baseline PET measures were negatively associated with subsequent PACC slopes within carriers (Aβ *r*_*s*_ = − 0.64, *p* = 0.03; PC tau *r*_*s*_ = − 0.68, *p* = 0.02; EC tau *r*_*s*_ = − 0.84, *p* = 0.002; IT tau *r*_*s*_ = − 0.84, *p* < 0.001). PACC decline rates were not associated with contemporaneous Aβ, EC tau or PC tau slopes (Aβ *r*_*s*_ = − 0.09, *p* = 0.78, Fig. 6e; EC *r*_*s*_ = − 0.23, *p* = 0.5; PC: *r*_*s*_ = − 0.36, *p* = 0.5), and were marginally associated with IT tau slopes (IT: *r*_*s*_ = − 0.63, *p* = 0.09; Fig. 6f) after multiple comparisons correction. Rates of PACC decline were not associated with baseline HV or HV slopes within carriers (both *p* > 0.6). We observed marginal associations in the expected direction between rates of decline in CERAD word list learning and baseline Aβ (*r*_*s*_ = − 0.48, *p* = 0.1) and HV slopes (*r*_*s*_ = 0.55, *p* = 0.07); all other associations with CERAD decline rates among carriers were non-significant (*p* > 0.2) after multiple comparisons correction.

## Discussion

Expanding upon previous cross-sectional findings, the longitudinal analyses in this study help to clarify the temporal sequence of the initiating pathological events in ADAD. Consistent with previous work in ADAD [40–44], the earliest biomarker changes we observed were in Aβ PET, with a global rate of increase of 4%/year seen as early as 16 EYO, followed by elevated entorhinal cortex (EC) tau as early as 9 EYO. Rapid increase in neocortical tau (inferior temporal cortex and precuneus) was observed in carriers closer to the time of symptom onset (1–6 EYO), neocortical tau change rates were associated with contemporaneous hippocampal volume loss (~ 6 EYO), and baseline tau levels were associated with subsequent cognitive decline (~ 4 EYO). These results are consistent with a trajectory of ADAD-related changes beginning with Aβ accumulation, followed by medial temporal tauopathy, followed by a cascade of neocortical tau accumulation, neurodegeneration, and cognitive decline.

Brain fibrillar Aβ accumulation associated with the *PSEN1*-E280A mutation is a steady process beginning decades prior to symptom onset [5, 6]. Consistent with previous PET studies of ADAD [40–43], we observed a clear separation between carriers and non-carriers in rates of Aβ PET change, which were elevated in 10 out of 12 carriers in our sample. Aβ change rates showed a negative quadratic relationship with baseline Aβ levels, consistent with previous work implying that Aβ follows a sigmoidal trajectory of accumulation [39]. As expected, the anatomy of cortical Aβ PET change in carriers was diffuse, and we observed the highest change rates in frontal, parietal, and lateral temporal cortices, consistent with other PET studies describing regional Aβ PET in ADAD [41, 44] and sporadic AD [45]. Supplementary analyses of regional Aβ (Supplementary Figure 4) suggested that precuneus or striatum PiB may capture earlier Aβ accumulation compared to the global measure, and that striatum PiB had the strongest associations with cross-sectional and longitudinal tau PET measures, consistent with previous studies [31, 32].

This is, to our knowledge, the first report assessing longitudinal rates of tau accumulation in ADAD in vivo using PET. Contemporaneous change rates of tau and Aβ PET were not correlated among carriers, consistent with the two pathologies being asynchronous. Among carriers who showed rapid neocortical tau increase, baseline Aβ burdens ranged from 1.3–2.2 DVR (~ 20–80 CL). These Aβ levels are all above thresholds generally considered to indicate abnormality (i.e., 14–20 CL), consistent with Aβ preceding tau accumulation in ADAD, as well as with findings from sporadic AD that Aβ appears to be a major driver of neocortical tau accumulation [16]. On the other hand, we observed considerable variability in rates of tau accumulation among carriers with similarly elevated Aβ burdens, and striatum Aβ was a stronger predictor of subsequent tau increase than cortical Aβ in this sample, suggesting that there are likely other factors in addition to cortical fibrillar Aβ pathology that trigger neocortical tau accumulation in ADAD. This is consistent with a recent report of a *PSEN1* E280A carrier who also carried the *APOE3* Christchurch mutation and, remarkably, was in her seventies with severely elevated neocortical Aβ, but relatively limited neocortical tau and preserved cognitive function [46].

We assessed tau PET primarily in three ROIs—EC, IT, and PC, representing MTL allocortex, temporal neocortex, and extra-temporal neocortex, respectively. EC tau levels were significantly elevated in all impaired carriers and in unimpaired carriers as early as 9 EYO—younger than we previously reported in this cohort [18]. IT and PC tau levels were elevated later (6 EYO), although still during the preclinical phase, in contrast to a previous report that tau PET is only elevated in ADAD cases with impairment [19]. Across ROIs, baseline EC tau levels predicted subsequent neocortical tau change rates but not vice-versa, consistent with MTL tauopathy occurring before tauopathy in temporal and extra-temporal neocortices. While this is consistent with the typical spatiotemporal progression of AD tauopathy implied by autopsy studies [17], some reports have suggested that EC tau may be less involved in ADAD compared to sporadic AD, as ADAD patients are younger and so are less likely to have age-related EC tauopathy observed in older individuals [19]. Our results suggest that EC tau plays an important role in ADAD: EC tau levels were predictive of subsequent neocortical tau increase and cognitive decline, supporting the idea that elevated EC tau PET signal is detectable at a relatively early disease stage [13, 47] and is a harbinger of future neocortical tau accumulation [15, 23, 48] and cognitive decline [13, 49].

We observed higher rates of tau change among carriers in neocortical regions compared to EC. This is consistent with studies in sporadic AD showing that cognitively impaired patients show lower rates of tau change in MTL compared to neocortex [15, 20, 21] and implies that MTL tau may reach a plateau at later disease stages while neocortical tau continues to increase. Notably, the rapid increase in neocortical tau PET we observed mirrors the observation that cerebrospinal fluid measures of soluble p-tau decrease around the time of symptom onset in ADAD [40, 50] and in more advanced sporadic AD cases [51]. Together with reports that CSF tau measures are consistently elevated in ADAD around 15 EYO [9, 10, 50], these results suggest that soluble p-tau levels initially increase in association with fibrillar Aβ pathology [52] and later decrease, possibly due to sequestration into neocortical neurofibrillary tangle pathology [40] and/or neuronal loss [50].

We note several differences between our longitudinal tau PET findings and what has been reported in sporadic AD. First, elevated tau PET levels were only observed in carriers with elevated neocortical Aβ PET (Fig. 1), whereas elevated tau without significant fibrillar Aβ burden has been frequently observed in both neuropathological [17] and PET studies of sporadic (preclinical) AD cases [15, 53]. Similarly, while autopsy and PET studies of sporadic AD have consistently observed greater tau burden in temporal neocortex than parietal neocortex [13, 54], implying a spatiotemporal progression of tau from medial temporal allocortex to temporal neocortex to extra-temporal neocortex, we observed elevated tau in PC (Braak V) in carriers without elevated tau in IT (Braak IV). This suggests that neocortical tau accumulation may proceed in a different anatomical sequence in ADAD compared to typical sporadic AD [19], although the initial involvement of EC tauopathy appears clear in both contexts. Recent longitudinal tau PET studies have highlighted the heterogeneity of tau accumulation patterns in sporadic AD [55, 56], and the heterogeneity we observed among carriers in this sample suggests that this will be an important factor to keep in mind for future work in ADAD.

The rates of neocortical tau increase we observed here (PC mean: 0.16 SUVR/year, 9%/year; max: 0.46 SUVR/year, 28%/year) were markedly higher than has been reported in sporadic AD (~ 0.05 SUVR/year, 3–6%/year) [20–22], although one study of sporadic AD reported rates as high as 8%/year tau PET increase in Braak V (including precuneus) [24]. In previously reported longitudinal PET data from our group processed with the same pipeline reported herein [15], sporadic AD patients showed rates of tau change in precuneus (mean ± SD [range]) of 0.091 ± 0.157 [− 0.057 0.448] SUVR/year, or on average 3.7% per year. These findings are consistent with early neuropathological reports suggesting that some ADAD mutations, including *PSEN1* E280A, may be associated with more rapid accumulation of neurofibrillary tangle pathology compared to sporadic AD [7]. However, both ADAD and sporadic AD cohorts show substantial variability in rates of tau accumulation, and direct, systematic comparison of these groups modeling for various confounds should be pursued in future work.

Consistent with previous observations [9, 10, 40, 43], we observed hippocampal atrophy in unimpaired carriers nearing the time of symptom onset, as early as 6 EYO. HV change rates were highly correlated with tau change rates but not Aβ change rates, suggesting that hippocampal atrophy occurs in temporal proximity to neocortical tau accumulation. This is consistent with findings from sporadic AD, where it has been reported that neurodegeneration is more closely associated with tau than with Aβ [21]. Whereas we focused our analyses on proxy variables for tau PET and structural measurements in this study, future studies of local associations between tau PET and atrophy in ADAD may help to elucidate the extent to which neurodegeneration is mediated by coincident (local) tau pathology.

Finally, we measured changes in cognitive performance over time and observed that rates of cognitive decline were correlated with baseline age, Aβ level, and all tau measures within carriers. Steep cognitive decline (> 0.2 *z*-score/year) was observed within carriers as early as 4 EYO with the PACC, comparable to previous reports in ADAD assessing change in a composite cognitive measure [40]. PACC slopes were more closely associated with contemporaneous neocortical tau slopes than with Aβ slopes, although these associations did not reach statistical significance within carriers, probably due to the small sample size. These results suggest that there may be some temporal offset between neocortical tau accumulation and cognitive decline in ADAD, and raises the possibility that intervening against tauopathy even in the context of elevated Aβ could be of therapeutic benefit.

### Limitations

This study has several limitations. First, our small sample size likely limited our ability to detect some significant effects, meaning that null findings, including those trending toward significance, may be significant with greater power. Similarly, whereas we limited the present study to hypothesis-driven analyses to limit the number of statistical comparisons, future studies with larger samples will be able to employ data-driven methods to comprehensively explore imaging and cognitive measures. For instance, previous studies have suggested that specific composite ROIs for structural MRI measurements may reflect AD-related neurodegeneration better than a single HV measure [57], whereas we focused the present analyses on HV. Our longitudinal PET findings require confirmation in other ADAD cohorts, as we only examine one ADAD mutation here. Finally, PET results can be influenced by the method of partial volume correction and choice of reference region [58], and although we found that the use of PVC did not appear to systematically bias our findings (Supplementary Figure 5), we did not undertake a systematic evaluation of the impact of various PET processing parameters. Studies using alternative processing methods and/or PET ligands may provide important opportunities to validate our findings.

## Conclusions

In summary, this study expands on previous cross-sectional findings by tracking the longitudinal relationships between Aβ, tau, neurodegeneration, and cognitive decline in ADAD. Our observations were consistent with a progression of pathologic changes beginning with Aβ accumulation and followed by entorhinal tau, neocortical tau, neurodegeneration, and cognitive decline. Notably, entorhinal tau accumulation is observable with PET years before symptom onset despite the young age of this ADAD cohort, and entorhinal tau burden predicts subsequent neocortical tau accumulation and cognitive decline in those with elevated Aβ burden. These findings have important implications for sporadic AD and suggest that entorhinal tau may act as a link between Aβ accumulation and catastrophic neocortical tau spread in both contexts.

## Supplementary Information

**Additional file 1: Supplementary Figure 1.** Mean images of PiB DVR and FTP SUVR for each group at baseline and follow-up.

**Additional file 2: Supplementary Figure 2.** Comparison of biomarker slopes derived from ordinary least squares (OLS) and linear mixed-effects (LME) models.

**Additional file 3: Supplementary Figure 3.** Regional accumulation of Aβversus age.

**Additional file 4: Supplementary Figure 4.** Assessment of striatum and precuneus Aβ predicting tau accumulation.

**Additional file 5: Supplementary Figure 5.** Impact of partial volume correction on PET results.

## Data Availability

Anonymized clinical, genetic, and imaging data are available upon request, subject to an internal review by YTQ and FL to ensure that the participants’ anonymity, confidentiality, and *PSEN1* E280a carrier or non-carrier status are protected. Data requests will be considered based on a proposal review, and completion of a data sharing agreement, in accordance with the University of Antioquia and MGH institutional guidelines. Please submit data requests to Y.T.Q.
