# Supplementary material for: Longitudinal amyloid and tau accumulation in autosomal dominant Alzheimer’s disease: findings from the Colombia-Boston (COLBOS) biomarker study
Source: Alzheimers Res Ther. 2021 Jan 15;13:27. doi: 10.1186/s13195-020-00765-5 (PMC7811244; doi:10.1186/s13195-020-00765-5)
Supplement: Supplementary file 1 — Additional file 1: Supplementary Figure 1. Mean images of PiB DVR and FTP SUVR for each group at baseline and follow-up. [file 13195_2020_765_MOESM1_ESM.docx]

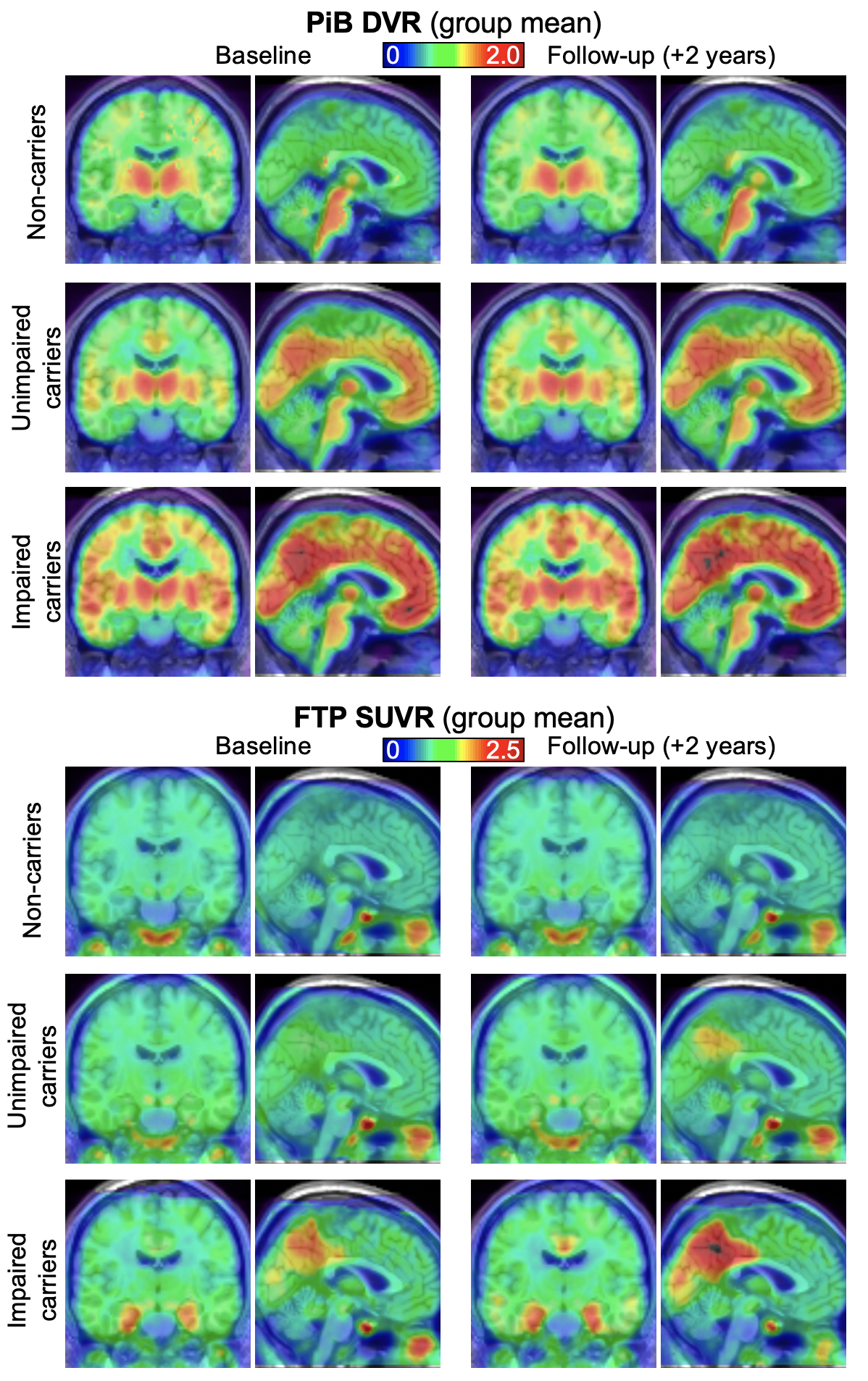


**Supplementary Figure 1**. **Mean images of PiB DVR (*top*) and FTP SUVR (*bottom*) for each group at baseline (*left*) and follow-up (*right*).**
