# Supplementary material for: Longitudinal amyloid and tau accumulation in autosomal dominant Alzheimer’s disease: findings from the Colombia-Boston (COLBOS) biomarker study
Source: Alzheimers Res Ther. 2021 Jan 15;13:27. doi: 10.1186/s13195-020-00765-5 (PMC7811244; doi:10.1186/s13195-020-00765-5)
Supplement: Supplementary file 2 — Additional file 2: Supplementary Figure 2. Comparison of biomarker slopes derived from ordinary least squares (OLS) and linear mixed-effects (LME) models. [file 13195_2020_765_MOESM2_ESM.docx]

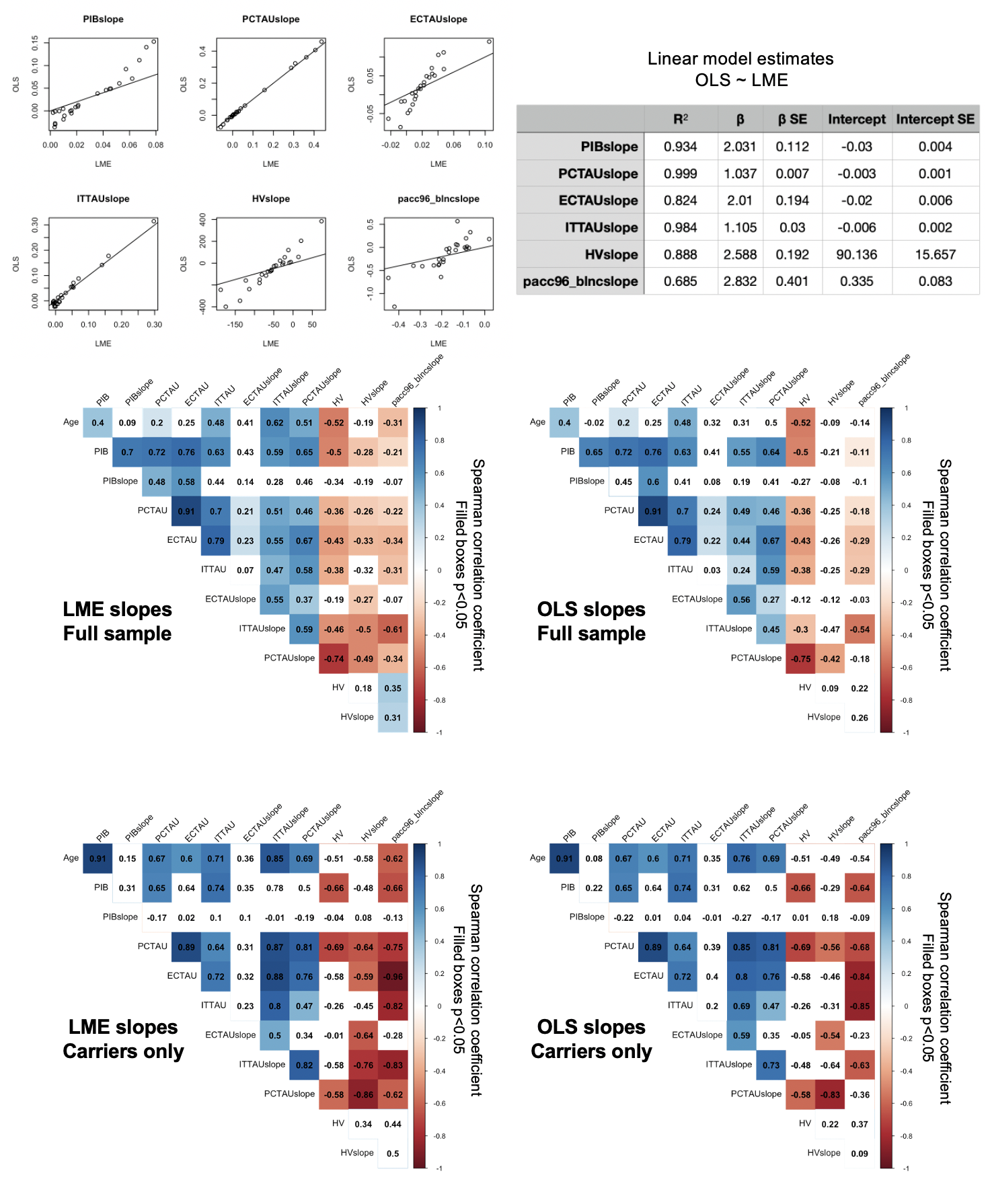


**Supplementary Figure 2. Comparison of biomarker slopes derived from ordinary least squares (OLS) and linear mixed-effects (LME) models.** *Top left*, scatter plots showing relationship between slopes derived from LME (X-axis) and OLS (Y-axis) for all slope variables, with line of unity in black. *Top right*, table summarizing outputs for bivariate linear models of LME versus OLS PET variables. We found that, as expected, the slopes extracted from LME and from OLS models were highly correlated; the choice of method appeared to have more of an impact on PACC, hippocampal volume, and entorhinal tau slopes compared to the other measures. *Bottom*, correlation matrices show top-line findings (i.e., correlations between PET and other variables in this study) for LME- (*left*) and OLS-derived (*right*) slopes, in the full sample (*top*) and in carriers only (*bottom*). Value of each cell is the Spearman correlation coefficient, indicated also by color scale; cells filled with color were significant at p<0.05. Findings were consistent whether slopes were extracted from LME or OLS models. Correlations with biomarker slope variables were slightly stronger when using slopes extracted from LME compared to OLS models, but not enough to change our understanding of the results or conclusions.
