# Supplementary material for: Longitudinal amyloid and tau accumulation in autosomal dominant Alzheimer’s disease: findings from the Colombia-Boston (COLBOS) biomarker study
Source: Alzheimers Res Ther. 2021 Jan 15;13:27. doi: 10.1186/s13195-020-00765-5 (PMC7811244; doi:10.1186/s13195-020-00765-5)
Supplement: Supplementary file 3 — Additional file 3: Supplementary Figure 3. Regional accumulation of Aβversus age. [file 13195_2020_765_MOESM3_ESM.docx]

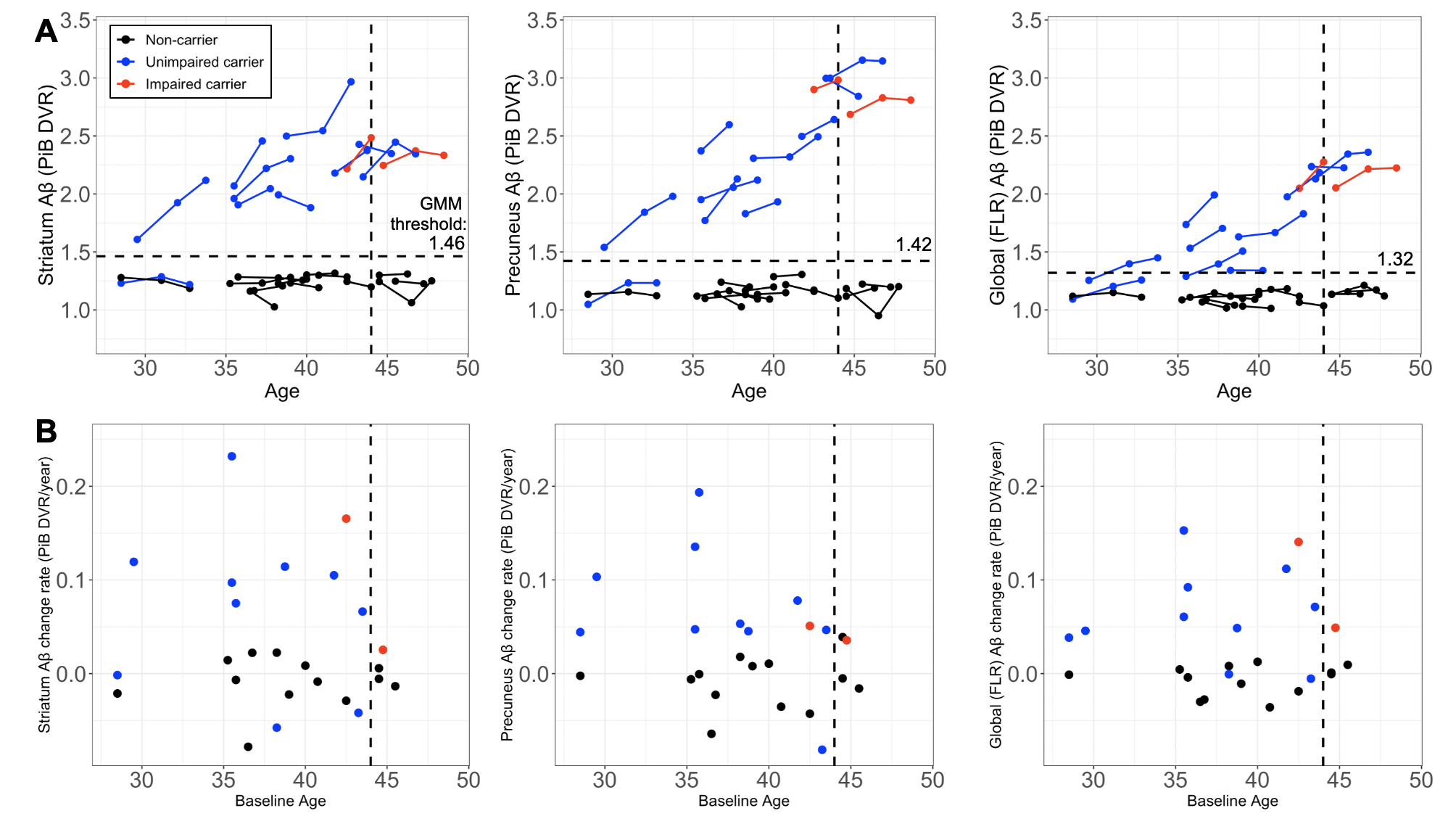


**Supplementary Figure 3. Regional accumulation of Aβ versus age.** (A) Spaghetti plots showing individual participants’ PiB DVR measurements over time in striatum (left), precuneus (center), and global (right) regions of interest. Horizontal dashed lines and inset text indicate region-specific thresholds derived from Gaussian mixture models; vertical dashed line indicates the expected age of MCI onset for carriers in this cohort. (B) Rates of Aβ accumulation as PiB DVR/year in each region as in (A). Data points are colored according to carrier status and cognitive status, per legend in top left.
