# Supplementary material for: Longitudinal amyloid and tau accumulation in autosomal dominant Alzheimer’s disease: findings from the Colombia-Boston (COLBOS) biomarker study
Source: Alzheimers Res Ther. 2021 Jan 15;13:27. doi: 10.1186/s13195-020-00765-5 (PMC7811244; doi:10.1186/s13195-020-00765-5)
Supplement: Supplementary file 4 — Additional file 4: Supplementary Figure 4. Assessment of striatum and precuneus Aβ predicting tau accumulation. [file 13195_2020_765_MOESM4_ESM.docx]

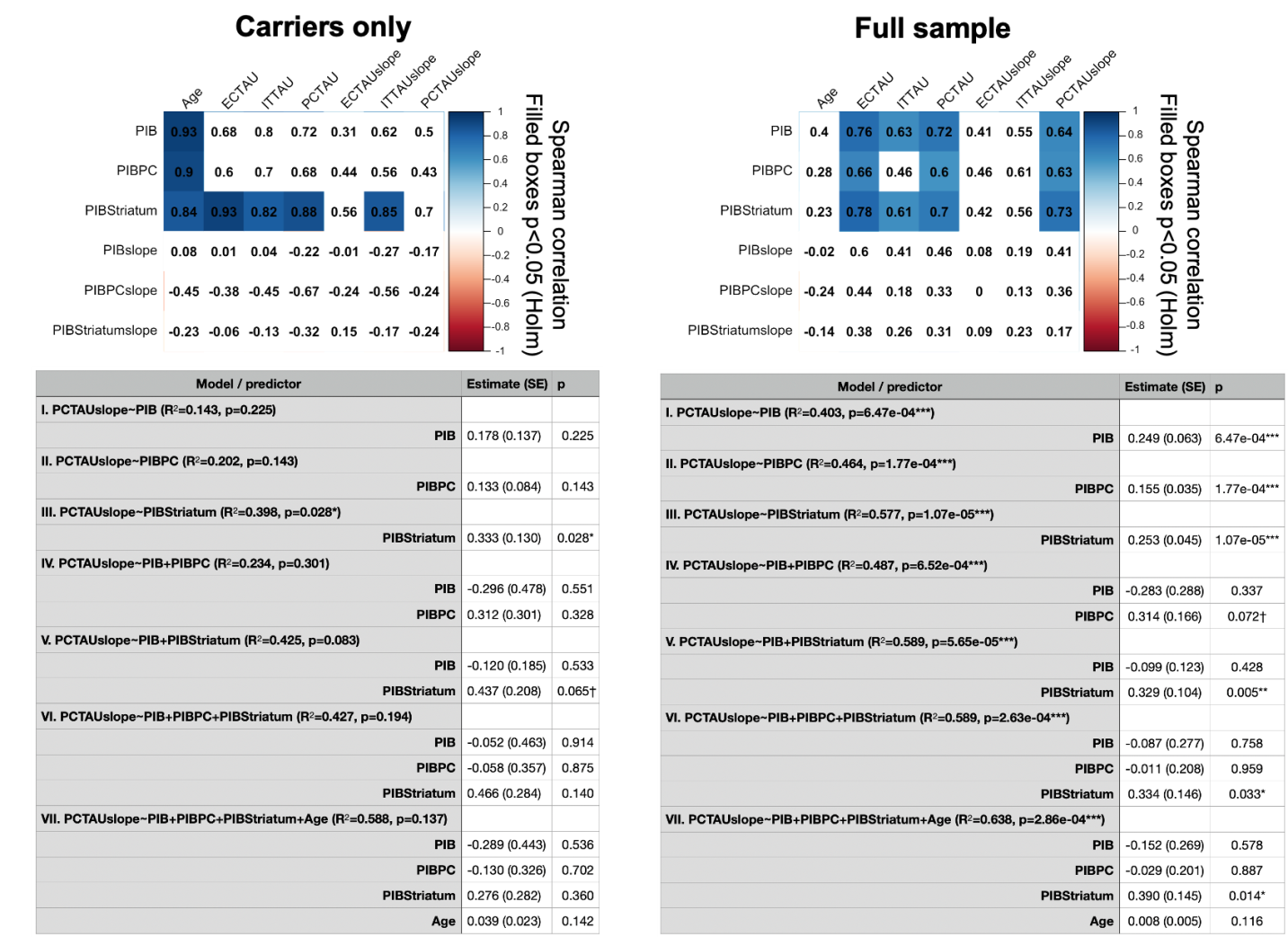


**Supplementary Figure 4. Assessment of striatum and precuneus Aβ predicting tau accumulation.** We assessed baseline and slope measurements of PiB DVR in precuneus and striatum (representing two regional measures that may capture earlier Aβ accumulation in ADAD) and their relationships with FTP PET baseline and slope measures. Within carriers, striatum PiB showed stronger associations with baseline FTP and FTP slopes compared to global or precuneus PiB (top left). The same was true when the analyses were repeated using the full sample (top right).

To test whether one of these regions more strongly predicted subsequent FTP PET increase than the others, we also performed hierarchical regression of the three baseline PiB measurements predicting precuneus tau slope, both in the full sample and in carriers only (summarized in tables). These analyses revealed that striatum PiB captured more of the variance in FTP PET change rates compared to either global or precuneus PiB (Model III versus Models I-II). In the model with all three PiB measures competing in the full sample (Model VI), striatum PiB was the only predictor to explain unique variance over and above the other two (F-test p=0.03). The inclusion of age in this model did not significantly impact these results (below, Model VII). Taken together, these results indicate that striatum PiB may be more of a “driver” of neocortical tau accumulation compared to precuneus or global PiB burden.

PIB=global PiB; PIBPC=precuneus PiB; PIBStriatum=striatum PiB; EC=entorhinal cortex; IT=inferior temporal cortex; PC=precuneus; SE=standard error.
