# Supplementary material for: Longitudinal amyloid and tau accumulation in autosomal dominant Alzheimer’s disease: findings from the Colombia-Boston (COLBOS) biomarker study
Source: Alzheimers Res Ther. 2021 Jan 15;13:27. doi: 10.1186/s13195-020-00765-5 (PMC7811244; doi:10.1186/s13195-020-00765-5)
Supplement: Supplementary file 5 — Additional file 5: Supplementary Figure 5. Impact of partial volume correction on PET results. [file 13195_2020_765_MOESM5_ESM.docx]

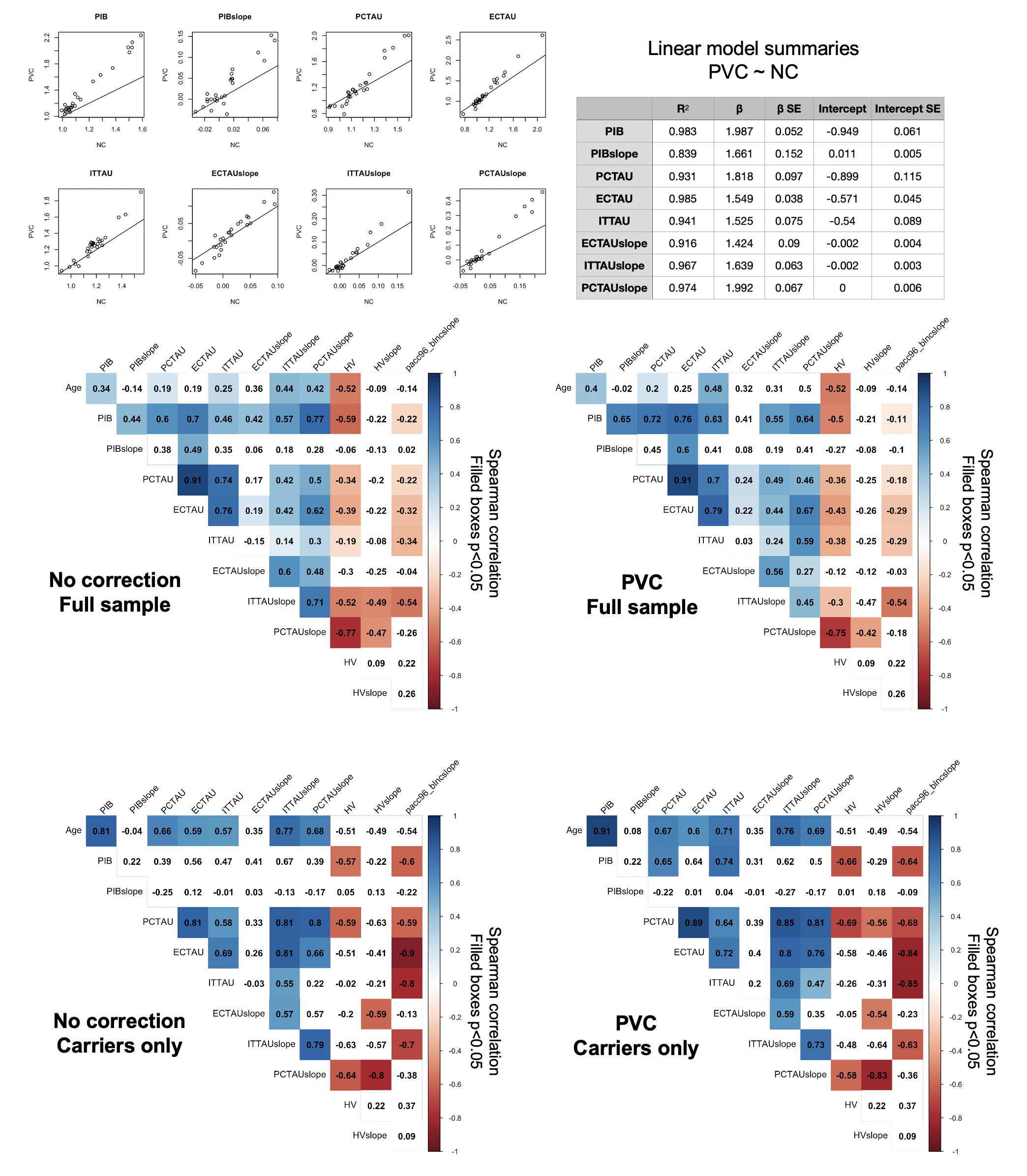


**Supplementary Figure 5. Impact of partial volume correction on PET results.** *Top left*, scatter plots showing uncorrected (NC, x-axis) versus partial volume corrected (PVC, y-axis) values for PET variables indicated in figure titles, with line of unity. *Top right,* table summarizing outputs for bivariate linear models of PVC versus NC PET variables. While PVC increased the dynamic range of both baseline and slope PET variables, NC and PVC values were highly correlated (R^2^>0.83). *Bottom*, correlation matrices show top-line findings (i.e., correlations between PET and other variables in this study) for NC (*left*) and PVC (*right*) PET data, in the full sample (*top*) and in carriers only (*bottom*). Value of each cell is the Spearman correlation coefficient, indicated also by color scale; cells filled with color were significant at p<0.05. While correlation coefficients varied slightly before and after PVC, the results were consistent and suggest that PVC did not introduce systematic bias that would have affected our results or conclusions. EC=entorhinal cortex; IT=inferior temporal cortex; PC=precuneus. PIB=global PiB DVR.
